# Evaluation of Genetic Mutations Associated with *Mycobacterium tuberculosis* Resistance to Amikacin, Kanamycin and Capreomycin: A Systematic Review

**DOI:** 10.1371/journal.pone.0033275

**Published:** 2012-03-29

**Authors:** Sophia B. Georghiou, Marisa Magana, Richard S. Garfein, Donald G. Catanzaro, Antonino Catanzaro, Timothy C. Rodwell

**Affiliations:** 1 Department of Molecular Biology, The Scripps Research Institute, La Jolla, California, United States of America; 2 University of California San Diego Medical Center, San Diego, California, United States of America; 3 Division of Global Public Health, University of California San Diego School of Medicine, La Jolla, California, United States of America; 4 Department of Family and Preventive Medicine, University of California San Diego Health Services Research Center, La Jolla, California, United States of America; 5 University of California San Diego Medical Center, San Diego, California, United States of America; 6 Division of Global Public Health, University of California San Diego School of Medicine, La Jolla, California, United States of America; National Institute of Allergy and Infectious Disease, United States of America

## Abstract

**Background:**

Rapid molecular diagnostics for detecting multidrug-resistant and extensively drug-resistant tuberculosis (M/XDR-TB) primarily identify mutations in *Mycobacterium tuberculosis* (*Mtb*) genes associated with drug resistance. Their accuracy, however, is dependent largely on the strength of the association between a specific mutation and the phenotypic resistance of the isolate with that mutation, which is not always 100%. While this relationship is well established and reliable for first-line anti-TB drugs, rifampin and isoniazid, it is less well-studied and understood for second-line, injectable drugs, amikacin (AMK), kanamycin (KAN) and capreomycin (CAP).

**Methodology/Principal Findings:**

We conducted a systematic review of all published studies evaluating *Mtb* mutations associated with resistance to AMK, KAN, CAP in order to characterize the diversity and frequency of mutations as well as describe the strength of the association between specific mutations and phenotypic resistance in global populations. Our objective was to determine the potential utility and reliability of these mutations as diagnostic markers for detecting AMK, KAN and CAP resistance. Mutation data was reviewed for 1,585 unique clinical isolates from four continents and over 18 countries. Mutations in the *rrs*, *tlyA*, *eis* promoter and *gidB* genes were associated with AMK, KAN and/or CAP resistance.

**Conclusions/Significance:**

The *rrs* A1401G mutation was present in the majority of AMK, KAN and CAP resistant *Mtb* strains reviewed, but was also found in 7% of CAP susceptible strains. The 1401 mutation alone, however, was not found with sufficient frequency to detect more than 70–80% of global *Mtb* strains resistant to AMK and CAP, and 60% of strains resistant to KAN. Additional mutations in the *rrs*, *eis* promoter, *tlyA* and *gidB* genes appear to be associated with resistance and could improve sensitivity and specificity of future diagnostics.

## Introduction

In 2010, 8.8 million new cases of tuberculosis (TB) and 1.4 million TB-related deaths were reported worldwide [1]. While the global incidence of TB appears to be decreasing, the emergence of multidrug-resistant TB (MDR-TB) and extensively drug-resistant TB (XDR-TB) has significantly complicated TB eradication efforts. MDR-TB strains, as defined by World Health Organization (WHO), are *Mycobacterium tuberculosis* (*Mtb*) strains resistant to the most effective “first-line” TB medications: isoniazid (INH) and rifampin (RIF). XDR-TB strains are characterized by resistance to INH, RIF (ie MDR-TB) plus any fluoroquinolone (e.g., moxifloxacin), and at least one of the three “injectable” anti-TB drugs: amikacin (AMK), kanamycin (KAN), and capreomycin (CAP) [2]. At an estimated 5% of all tuberculosis cases globally, the incidence of MDR-TB recently reported by the WHO is the highest ever recorded [3], and XDR-TB cases have been confirmed in 58 countries to date [4].

Appropriate use of the “second-line” injectable drugs, AMK, KAN and/or CAP is critical to the effective treatment of MDR-TB and to the prevention of XDR-TB. It is therefore imperative that the MDR-TB strains in patients starting second-line drug treatment are first tested for sensitivity to these drugs to ensure appropriate treatment choices are made and that resistance is not further amplified [5]. Conventional diagnosis of drug-resistance in *Mtb* strains relies heavily upon mycobacterial culture and drug susceptibility testing in liquid or solid media. While this method is effective for detecting INH and RIF resistance, detecting resistance to AMK, KAN and CAP is more complicated and less reliable [6]. Additionally, results are only obtained after weeks to months of incubation and many developing countries lack the resources to establish the stringent laboratory conditions needed for these growth-based methods. From a clinical perspective, the existing growth-based diagnostics also reveal too little, too late, as patients undergoing treatment with drugs to which they are resistant, remain contagious, and those with XDR-TB and HIV often die before they are even diagnosed [7]. Rapid molecular diagnostic tests that do not rely upon the culture of this slow-growing pathogen are essential to the future management of M/XDR-TB.

Molecular diagnostics, which focus on the detection of mutations in pathogen genes that have been associated with drug resistance, have shown the most promise for rapid diagnosis of M/XDR-TB [8]–[10]. Their accuracy, however, is dependent largely on the strength of the association between a specific *Mtb* gene mutation and the phenotypic resistance of the isolate with that mutation, which is not always 100%. This relationship between mutation and phenotypic resistance is well established and consistent for the first-line drug RIF [11], [12], and to a lesser extent INH [13], [14], but it is less well-studied and understood for the injectable drugs.

To date, the primary genes associated with injectable drug resistance in *Mtb* have been identified based on an understanding of the mechanisms of action of the aminoglycosides (AMK, KAN) and cyclic peptides (CAP) against *Mtb*. Both AMK and KAN bind to the 16S rRNA in the 30S ribosomal subunit and inhibit protein synthesis [15]. The mechanism of action of CAP in *Mtb* is not well understood, but it appears to interfere with translation and inhibit phenylalanine synthesis in mycobacterial ribosomes [16]. Mutations in *Mtb*, mostly non-synonymous, single nucleotide polymorphisms (SNPs), that prevent the binding of the injectable drugs to their pathogen gene targets or that change the mechanism of action of the drugs, have been associated with drug resistance to AMK, KAN and CAP [17], [18]. The most well-studied gene mutations believed to confer resistance to the injectable drugs lie in the *rrs* and *tlyA* genes. The *rrs* gene encodes 16S rRNA [19] and *tlyA* encodes a 2′-O-methyltransferase that modifies nucleotide C1409 in helix 44 of 16S rRNA and nucleotide C1920 in helix 69 of 23S rRNA [20]. The mutations most commonly reported to cause resistance to the injectable drugs include: A1401G, C1402T, and G1484T in the *rrs* gene [9], [10], [18], [21]–[38]. More recently, the *eis* promoter, which has been demonstrated to enhance the intracellular survival of a related bacterium—*Mycobacterium smegmatis*—has also been considered for its utility as a marker for resistance to KAN when mutated [39]. Other resistance-associated mutations in these genes and other genes have been proposed [32], but their utility as accurate predictors of resistance has yet to be demonstrated. Consequently, current molecular detection tests for XDR-TB are considered inferior to those used for MDR-TB due to insufficient knowledge about the key mutations responsible for conferring resistance to AMK, KAN and CAP.

We therefore conducted a systematic review of all published studies evaluating *Mtb* mutations associated with resistance to AMK, KAN, CAP in order to characterize the diversity and frequency of mutations as well as describe the strength of the association between specific mutations and phenotypic resistance in global populations. The objective of our study was to determine the potential utility and reliability of these mutations as diagnostic markers for detecting AMK, KAN and CAP resistance in *Mtb*.

## Methods

### Literature Search

A Medline search was conducted of all publications evaluating mutations associated with resistance to AMK, KAN and CAP in *Mtb*. The search was restricted to studies published from 1959 through September 10^th^, 2011, including those studies available online prior to publication. MEDLINE/PubMed key search terms used were: (second-line OR kanamycin OR capreomycin OR amikacin) AND (resistance OR resistant) AND tuberculosis AND (mutation OR sequence OR gene).

### Study Selection Criteria

Studies were included if they met the following predetermined criteria: i) published in English; ii) presented original data; and iii) assessed drug resistance mutations in clinical *Mtb* strains that were resistant to AMK, KAN and/or CAP (*in vitro* studies were excluded as laboratory generated mutations have been frequently observed to be different from those found in clinical isolates [40]). Studies were also excluded if they did not perform or describe phenotypic drug susceptibility testing (DST), did not perform sequencing as a method for determining drug resistance mutations, or did not report specific mutation data. We included studies that used both liquid and solid-based media for DST as long as they reported clearly defined drug concentration cutoffs for determining resistance. These criteria were intended to select the broadest range of studies with the highest quality of specific data for mutation analysis.

### Data Extraction and Entry

From each publication, we extracted the following information: authors, publication year, geographic origin of clinical strains, reference strain(s) utilized to evaluate mutations, testing method for phenotypic drug susceptibility and drug concentrations used for defining resistance, methods used to detect genotypic mutations, genes sequenced, and loci of genes sequenced. The following mutation information was also recorded: specific gene mutation(s) found, injectable drug utilized for selection, number of resistant and susceptible isolates tested, and number of resistant and susceptible isolates demonstrating mutation. Data were recorded and compiled using Excel and Access software (Microsoft, Redmond, WA).

### Data Collation and Cumulative Mutation Frequency Calculations

Mutations associated with resistance to AMK, KAN, and CAP were grouped by genes and stratified by the drug resistance phenotype associated with the mutation. Data was examined for evidence of individual as well as multiple mutations within a gene. Each mutation reported in a resistant *Mtb* isolate was considered independent of all others within and between studies (except where otherwise noted for multiple mutations in the same gene), and recorded as one instance of the mutation in the numerator of our cumulative frequency calculations. Cumulative mutation frequency in *resistant* isolates was calculated as the number of resistant isolates in which the mutation was found, divided by the total number of phenotypically resistant isolates tested across studies. Cumulative mutation frequency in *susceptible* isolates was calculated as the number of susceptible isolates in which the mutation was found, divided by the total number of susceptible isolates tested across studies. As not all studies examined all mutations or all genes associated with resistance, an isolate was only included in the denominator of a cumulative frequency calculation for a particular mutation if that mutation could have been detected in that isolate (i.e. the study sequenced the appropriate section of the gene).

While all mutations identified in the review were evaluated, the cumulative mutation frequency tables presented in this review represent only the mutations that reached a frequency threshold as described below. Isolates with *rrs* and *gidB* gene mutations were included if their frequency among resistant isolates was at least 1% for any one of the injectable drugs tested, and when that frequency was *not* higher among susceptible isolates than resistant isolates for two or more injectable drugs. The isolates with *tlyA* gene, *eis* promoter, and multiple *rrs* mutations were included in the tables if the mutation frequency among resistant isolates was at least 1% for any one of the injectable drugs tested. Mutations were excluded from the tables when the frequencies of the mutation were equal in resistant and susceptible strains (ie not likely to be associated with resistance).

### Quality Assessment and Consistency

Throughout this review mutation co-ordinates are referred to in accordance with the numbering in the *Mtb* H37Rv complete genome, accession number NC_000962, National Center for Biotechnology Information [41]. Mutations identified in earlier publications that did not use this co-ordinate system [21], [22], [25] were updated to this numbering system. The H37Rv strain was the assumed template for mutation analysis in all publications, even when not explicitly stated within a given study, as is standard practice [9], [10], [18], [25], [26], [30].

In publications that did not examine the whole gene and did not report the specific area of the gene sequenced, the reported SNPs were substituted as the outer limits for the gene region under study (e.g. if only *rrs* mutations A1401G and G1484T were reported within a publication, the sequence from 1401–1484 was considered to be the section of *rrs* analyzed for mutations). In publications evaluating the MTBDR*sl* line probe assay (Hain Life Sciences, Tübingen, Germany), in which resistance-associated genes were sequenced, but sequence data was only provided for regions hybridized to the line probes, only those SNPs tested via hybridization strips were considered sequenced.

In one publication, mutation data was reported for “probable-resistant” and “probable-susceptible” clinical isolates [33]. A “probable-susceptible” strain was defined as an *Mtb* strain isolated prior to initiation of any treatment with a given drug, while a “probable-resistant” strain was defined as a strain isolated from patients after at least six months of treatment with the given drug. For the purposes of this review, we assumed the probable-resistant and probable-susceptible isolates were resistant and susceptible based on the DST methodology described in the manuscript.

### Sensitivity and Specificity of Mutations

In order to comment on the potential predictive value of the reported resistance-related mutations in a molecular diagnostic test, we described a mutation as potentially “very sensitive” when its cumulative frequency was 75% or more in the resistant strains evaluated and “moderately sensitive” if it occurred in 50% to 74% of the resistant strains reported. A resistance-related mutation was defined as “very specific” if it was reported in 5% or less of susceptible strains evaluated and “moderately specific” if it was reported in 6% to 10% of the susceptible strains evaluated.

## Results

### Description of Included Studies

**Figure 1:**
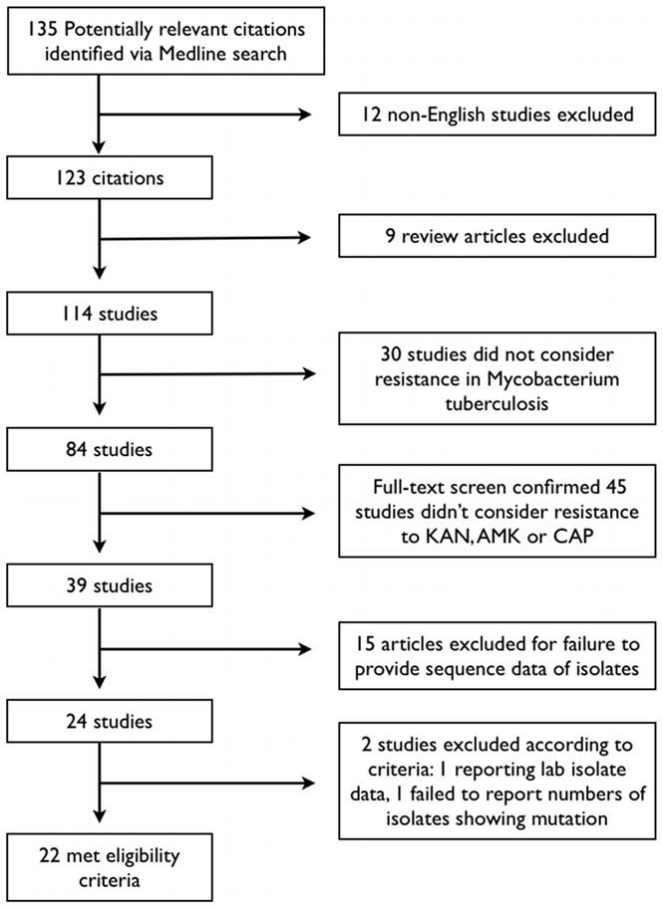
Study Selection Process and Reason for Exclusion of Studies.

Figure 1 (Figure S1) illustrates the study selection and exclusion process utilized for this review. Initial search parameters identified 135 studies published from November, 1959 through September, 2011. Twenty-two publications met all eligibility criteria and were included in the review [6]–[27].

Of the 22 studies included, the earliest was published in 1998 and 17 (77%) were published in the last three years (Table 1). Altogether, mutation data was provided for 1,585 unique clinical isolates with various phenotypic resistance profiles to AMK, KAN, and/or CAP. The reported geographic origins of these strains were diverse, covering four continents and over 18 countries.

**Table 1:** Details of Studies Included in Review and Source of *Mycobactrium tuberculosis* Isolates.

	Clinical Isolates			
PubMed ID	# of Clinical Isolates Examined	Origin of Isolates	Clinical Institution(s) Providing Isolates	Year of Collection
09574680	114	Japan	Various hospitals	Not stated
			Japan Anti-TB Association	
			Research Institute of TB	
			Hiroshima University	
09593173	17	Not stated	PHRI TB Center, New York	Not stated
12937004	49	Estonia	Not stated	2001
15673735	18	Not stated	CDC	Not stated
16048924	16	Not stated	CDC	Not stated
19906990	58	Not stated	CDC	Not stated
19386845	106	Germany	National Reference Laboratory	Not stated
19470506	87	Uzbekistan	Not stated	2003–2006
19634718	15	China	Beijing TB and Lung Tumor Research Institute	Not stated
19752274	145	Georgia	Georgian National Reference Laboratory	Not stated
19890396	11	South Africa	Not stated	1994, 1995, 2005, 2006
20028780	26	Portugal	Various hospitals and laboratories	2005
20032248	106	South Korea	National Masan Tuberculosis Hospital	Not stated
20185419	10	South Africa	Groote Schuur Hospital	2006, 2009
20335420	52	France	French Reference Center of Mycobacteria	2005–2009
20573868	62	Vietnam	Pham Ngoc Thach Hospital	2005–2006
20713679	6	India	Super Religare Laboratories Reference Center	Not stated
20854453	133	Korea	Korean Institute of Tuberculosis	Not stated
		USA	Massachusettes State Laboratory Institute	
		Philippines	Tropical Disease Foundation	
		Latvia	State Agency of Tuberculosis and Lung Diseases	
		China	Public Health Laboratory Centre	
21300839	314	Not stated	CDC	Not stated
21427106	152	Various Countries	Not stated	Not stated
21562102	38	Taiwan	Not stated	2008–2009
21732736	50	South Africa	National Health Laboratory Services	2008–2009

**CDC = Center for Disease Control and Prevention, Atlanta.**

Mutations in the *rrs*, *tlyA*, *eis* promoter and *gidB* genes were associated with *Mtb* resistance to AMK, KAN and/or CAP in the examined literature. A total of 94 unique mutations were reported relative to the reference H37Rv genome: *rrs* (37 unique mutations), *tlyA* (30 unique mutations), *eis* (12 unique mutations) and *gidB* (15 unique mutations). Eighty-two of these mutations (87%) were SNPs, six were deletions (one in *rrs*, two in *tlyA* and three in *gidB*), and six were insertions (two in *rrs* and four in *tlyA*). As resistance determination for the strains was crucial to our analysis, we evaluated the DST methods and drug-concentration cutoffs used in each study to define whether a strain was resistant or not. Table 2 shows the method of DST and cutoff concentrations used in each study as well as whether or not they conformed to published standards. The drug concentrations used in all but three studies [10], [26], [36] conformed to at least one national or international published standard. As DST standards are somewhat controversial and variable between countries, and the three studies in question were all conducted by national reference laboratories, we assumed their DST definition of resistance was correct for this review. A PRISMA checklist is provided as befitting a systematic review, to ensure integrity of the data (Table S1).

**Table 2:** Drug Susceptibility Testing (DST) Methods Employed in Publications.

		Upper Limit Drug Concentration used to determine resistance (µl/ml)			
PubMed ID	DST Method	AMK	KAN	CAP	DST Conforms to Published Standard
19386845	MGIT 960	1.0	ND	2.5	Yes [73]
19470506	MGIT 960	1.0	ND	2.5	Yes [73]
20713679	MGIT 960	1.0	4.0	2.5	Yes [73]
21732736	MGIT 960	1.0	ND	2.5	Yes [73]
20028780	BACTEC 460	1.0	5.0	1.25	Yes [6]
20335420	Lowenstein-Jensen	20*	20	20*	Yes* [74]
20573868	Lowenstein-Jensen	ND	20	ND	Yes [74]
20854453	Lowenstein-Jensen	ND	30	40	Yes [75]
19634718	Lowenstein-Jensen	20*	20	ND	Yes* [74]
19752274	Lowenstein-Jensen	40	30	40	Yes [73]
09593173	Lowenstein-Jensen	“standard”	“standard”	ND	Yes [75]
19890396	Lowenstein-Jensen	ND	20	ND	Yes [74]
12937004	Middlebrook 7H10	4.0	4.0	ND	Yes [6], [76]
20032248	Middlebrook 7H10	4.0	5.0	10	Yes [6], [73], [76]
15673735	Middlebrook 7H10	4.0	5.0	10	Yes [6], [73], [76]
16048924	Middlebrook 7H10	4.0	5.0	10	Yes [6], [73], [76]
19906990	Middlebrook 7H10	4.0	5.0	ND	Yes [6], [73], [76]
21300839	Middlebrook 7H10	4.0	5.0	10	Yes [6], [73], [76]
21427106	Middlebrook 7H10	1.0	4.0	8.0	Yes [77]
20185419	Middlebrook 7H11	ND	6.0	ND	Yes [73]
21562102	Middlebrook 7H11	6.0*	6.0	10	Yes* [73]
09574680	Ogawa Egg Medium	ND	ND	100	Yes [78]

* No publication found to validate DST concentration.

AMK = amikacin, KAN = kanamycin, CAP = capreomycin.

ND = not done.

### *rrs* Mutations Associated with Injectable Drug Resistance

**Figure 2:**
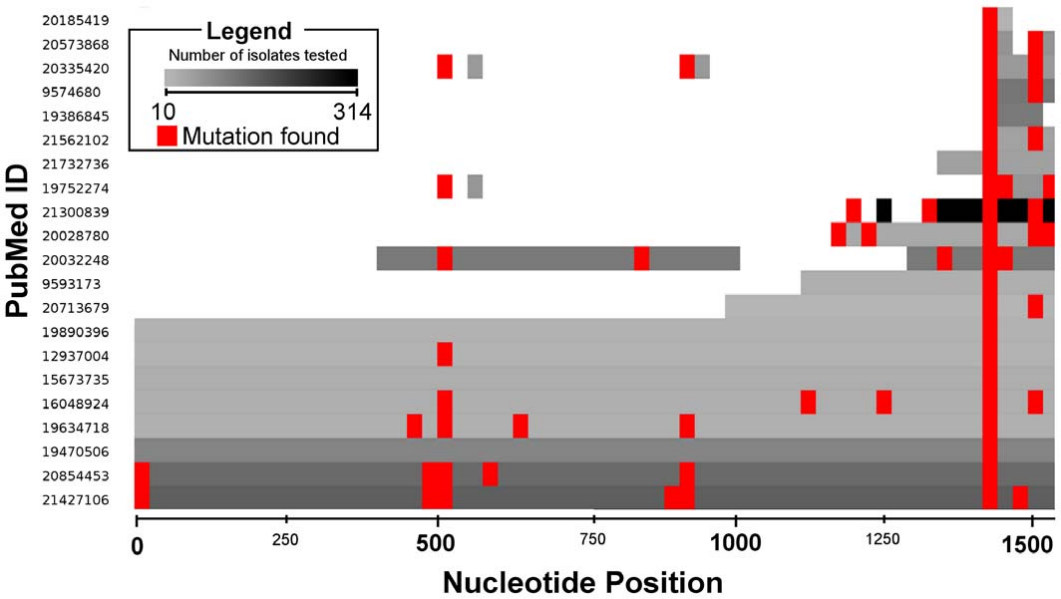
Heatmap of Reviewed Studies that Evaluated *rrs* Gene Mutations in *Mycobacterium tuberculosis* isolates. Graphic shows the region of the *rrs* gene studied, the number of isolates tested in each study and the locations of the mutations found. The X-axis (nucleotide position) has a 25 base pair resolution. The numbers of isolates varies from 314 (black) to 10 (lightest grey). Red indicates that a mutation has been found in that 25 base pair region.

Among the genes considered in the 22 papers reviewed, *rrs* was the most extensively studied, but only eight papers (36%), representing a total of 442 tested isolates (32% of the total), included sequence data on the entire *rrs* gene. Figure 2 shows the *rrs* studies as a heatmap of the number of isolates evaluated in each study as well as the locations of the major mutations found. The most commonly examined mutations were the A1401G mutation, reported in all 21 papers with *rrs* mutations, and the G1484T mutation, reported in 7/21 papers.

Table 3 shows the cumulative frequencies of the most commonly reported mutations in the *rrs* gene. The A1401G SNP was found in 387/493 (78%) of AMK-resistant (AMK^R^) strains and it was never observed in AMK-susceptible (AMK^S^) strains. It is notable that while 367/483 (76%) CAP-resistant (CAP^R^) isolates studied had the A1401G mutation, it was also found in 45/678 (7%) of CAP-susceptible (CAP^S^) strains tested across 7 studies. However only 354/637 (56%) KAN-resistant (KAN^R^) isolates studied had the A1401G mutation, and it was never reported in KAN-susceptible (KAN^S^) strains. These studies indicate that the A1401G mutation has potentially moderate sensitivity for predicting resistance to all three drugs and the potential to be a highly specific predictor for AMK and KAN resistance, but less so for CAP.

**Table 3:** Cumulative Frequencies of Selected Mutations within the *rrs* Gene among *Mycobacterium tuberculosis* Isolates Resistant or Susceptible to Amikacin (AMK), Kanamycin (KAN) and/or Capreomycin (CAP).

Mutation	Drug Tested	#R Examined	#S Examined	#R with Mutation	#S with Mutation	Frequency of Mutation among R	Frequency of Mutation among S
A1401G	AMK	493	703	387	0	0.78	0.00
	KAN	637	643	354	0	0.56	0.00
	CAP	483	678	367	45	0.76	0.07
A514C	AMK	200	263	20	15	0.10	0.06
	KAN	269	236	23	12	0.09	0.05
	CAP	195	273	14	14	0.07	0.05
C517T	AMK	200	263	14	10	0.07	0.04
	KAN	269	236	20	5	0.07	0.02
	CAP	195	273	8	14	0.04	0.05
A513C	AMK	196	260	1	1	0.01	0.00
	KAN	259	239	9	4	0.03	0.02
	CAP	190	271	1	1	0.01	0.00
G1484T	AMK	491	666	5	1	0.01	0.00
	KAN	616	654	9	0	0.01	0.00
	CAP	483	678	2	0	0.00	0.00
C1402T	AMK	443	624	3	6	0.01	0.01
	KAN	525	543	5	4	0.01	0.01
	CAP	434	637	7	0	0.02	0.00
G1158T	AMK	206	133	2	0	0.01	0.00
	KAN	273	116	2	0	0.01	0.00
	CAP	201	149	2	0	0.01	0.00
A907C	AMK	200	263	2	1	0.01	0.00
	KAN	259	236	2	1	0.01	0.00
	CAP	190	271	1	1	0.01	0.00
C1402	AMK	443	624	0	1	0.00	0.00
	KAN	525	543	3	0	0.01	0.00
	CAP	434	637	1	0	0.00	0.00

R = Resistant isolates.

S = Susceptible isolates.

Other notable *rrs* mutations included the A514C and C517T SNPs, which were found in only 7%–10% and 4%–7% of resistant strains, respectively. However, both mutations were also found in 2%–6% of isolates defined as susceptible to the injectable drugs. Surprisingly, while the G1484T and C1402T mutations are commonly cited as being strongly and commonly associated with resistance, our review found that out of over 400 injectable drug resistant strains evaluated, only 1% or less had these mutations. The C1402T appears to occur as frequently in AMK^S^ and KAN^S^ strains as it does in AMK^R^ and KAN^R^ strains suggesting it would be a poor marker of resistance. The *rrs* mutation G1158T, while studied in less than 300 injectable drug resistant strains, appears to be at least as frequent in resistant strains as the 1484 and 1402 *rrs* mutations, and has never been observed in the almost 150 strains susceptible to AMK, KAN and/or CAP, suggesting it might be at least as sensitive as 1484 and 1402 mutations and a more specific predictor for injectable drug resistance.

### *tlyA* Mutations Associated with Injectable Drug Resistance

Sequence data for the *tlyA* gene was provided in only 8 of the 22 publications reviewed [18], [24], [28], [31], [33], [35], [37], [38]. Mutations of the *tlyA* gene have been most well-studied in CAP^R^ and CAP^S^ *Mtb* strains (n = 366 and 559, respectively), and have been evaluated in less than 260 KAN^R^ or AMK^R^ strains (Table 4). Mutations in the *tlyA* gene associated with CAP^R^ were reported to be rare in the surveyed literature (found in ∼1–3% of resistant strains). However, when reported, they were not found in any CAP^S^ strains, making them potentially highly specific markers of CAP resistance. Of the 559 total CAP^S^ isolates reported in 7 studies [18], [24], [28], [31], [33], [35], [37], [38], none had any mutations within the *tlyA* gene. A GT insertion at position 755 of the *tlyA* gene was the only mutation that was found in more than 1% of CAP^R^ strains (3% of strains had this mutation).

**Table 4:** Cumulative Frequencies of Selected Mutations within the *tlyA* Gene among *Mycobacterium tuberculosis* Isolates Resistant or Susceptible to Amikacin (AMK), Kanamycin (KAN) and/or Capreomycin (CAP).

Mutation	Drug Tested	#R Examined	#S Examined	#R with Mutation	#S with Mutation	Frequency of Mutation among R	Frequency of Mutation among S
insGT755	AMK	257	221	2	9	0.01	0.04
	KAN	223	118	9	2	0.04	0.02
	CAP	366	559	11	0	0.03	0.00
G223T	AMK	257	184	2	0	0.01	0.00
	KAN	223	118	2	0	0.01	0.00
	CAP	366	559	2	0	0.01	0.00
insGC202	AMK	257	221	0	0	0.00	0.00
	KAN	223	118	0	0	0.00	0.00
	CAP	366	559	2	0	0.01	0.00
Gly196Glu*	AMK	257	221	0	0	0.00	0.00
	KAN	223	118	0	0	0.00	0.00
	CAP	366	559	2	0	0.01	0.00
T220C	AMK	257	221	0	0	0.00	0.00
	KAN	223	118	0	0	0.00	0.00
	CAP	366	559	2	0	0.01	0.00
T708G	AMK	257	221	0	3	0.00	0.01
	KAN	223	118	0	3	0.00	0.03
	CAP	366	559	3	0	0.01	0.00

* Represents an amino acid change, as specific nucleotide changes were not provided for this mutation.

R = Resistant isolates.

S = Susceptible isolates.

### *eis* Promoter Mutations Associated with Injectable Drug Resistance

Mutations within the *eis* promoter region of *Mtb* have only been evaluated in a few hundred injectable drug resistant strains (Table 5) and were reported to be largely associated with KAN resistance [37], [39]. The most frequently reported *eis* promoter SNP (G-10A) was found in 66/296 (22%) of the KAN^R^ strains studied—second only to the frequency of the *rrs* A1401G mutation in injectable drug resistant strains—and was found in only 2/266 (1%) of KAN^S^ strains. The C-14T SNP was reported in modest frequency in both KAN^R^ and AMK^R^ strains (11% and 9% respectively) and was not found in susceptible strains. *eis* promoter mutations were also reported in up to (9%) CAP^R^ strains. Aside from the C-12T SNP, however, which was reported in 5/93 (5%) of CAP^R^ strains and 1/93 (<1%) of CAP^S^ strains, these mutations appear to be non-specific markers of resistance, occurring in up to 29% of CAP^S^ strains.

**Table 5:** Cumulative Frequencies of Selected Mutations within the *eis* Promoter among *Mycobacterium tuberculosis* Isolates Resistant or Susceptible to Amikacin (AMK), Kanamycin (KAN) and/or Capreomycin (CAP).

Mutation	Drug Tested	#R Examined	#S Examined	#R with Mutation	#S with Mutation	Frequency of Mutation among R	Frequency of Mutation among S
G-10A	AMK	118	72	12	27	0.10	0.38
	KAN	296	266	66	2	0.22	0.01
	CAP	93	97	8	28	0.09	0.29
C-14T	AMK	118	72	11	0	0.09	0.00
	KAN	296	266	32	0	0.11	0.00
	CAP	94	97	5	6	0.05	0.06
C-12T	AMK	118	72	7	0	0.06	0.00
	KAN	296	266	9	6	0.03	0.02
	CAP	93	97	5	1	0.05	0.01
G-37T	AMK	118	72	2	0	0.02	0.00
	KAN	296	266	15	0	0.05	0.00
	CAP	93	97	0	2	0.00	0.02
G-10C	AMK	118	72	1	0	0.01	0.00
	KAN	296	266	1	0	0.00	0.00
	CAP	93	97	1	0	0.01	0.00
G-6T	AMK	118	72	1	0	0.01	0.00
	KAN	296	266	1	0	0.00	0.00
	CAP	93	97	1	0	0.01	0.00
C-15G	AMK	118	72	1	0	0.01	0.00
	KAN	296	266	1	0	0.00	0.00
	CAP	93	97	0	1	0.00	0.01

R = Resistant isolates.

S = Susceptible isolates.

### *gidB* Mutations Associated with Injectable Drug Resistance

The reported *gidB* mutations associated with injectable drug resistance have only been evaluated in 20 strains resistant to AMK, KAN and/or CAP (Table 6), producing what are likely unreliable cumulative frequencies. The G102 deletion appeared with a high frequency among resistant isolates (17–20%), and was only found in one susceptible isolate examined. The T230C, C286T, and T104G SNPs were each reported in one injectable drug resistant strain and did not appear to occur in strains susceptible to AMK, KAN and/or CAP.

**Table 6:** Cumulative Frequencies of Selected Mutations within the *gidB* Gene among *Mycobacterium tuberculosis* Isolates Resistant or Susceptible to Amikacin (AMK), Kanamycin (KAN) and/or Capreomycin (CAP).

Mutation	Drug Tested	#R Examined	#S Examined	#R with Mutation	#S with Mutation	Frequency of Mutation among R	Frequency of Mutation among S
ΔG102*	AMK	17	89	3	1	0.18	0.01
	KAN	20	86	3	1	0.15	0.01
	CAP	18	88	3	1	0.17	0.01
T230C	AMK	17	89	1	0	0.06	0.00
	KAN	20	86	1	0	0.05	0.00
	CAP	18	88	1	0	0.06	0.00
C286T	AMK	17	89	1	0	0.06	0.00
	KAN	20	86	1	0	0.05	0.00
	CAP	18	88	1	0	0.06	0.00
T104G	AMK	17	89	1	0	0.06	0.00
	KAN	20	86	1	0	0.05	0.00
	CAP	18	88	1	0	0.06	0.00
A254G	AMK	17	89	1	1	0.06	0.01
	KAN	20	86	1	1	0.05	0.01
	CAP	18	88	1	1	0.06	0.01

All mutations in this table represent mutations found via sequence analysis in just one study [32].

* Δ Represents nucleotide deletion. SNPs were grouped for brevity.

R = Resistant isolates.

S = Susceptible isolates.

### Double Mutations in the *rrs* Gene Associated with Injectable Drug Resistance

Only a small number of the surveyed publications described specific mutations at more than one site in any of the genes evaluated, limiting our ability to calculate cumulative frequencies of such “multiple mutations” in injectable drug resistant strains. We therefore report here only specific double mutations within the most commonly described gene (*rrs*). The most common double mutations reported always included the A1401G mutation (Table 7). While the cumulative frequency of double mutations did not exceed 5% in injectable drug resistant strains, an A1401G mutation together with A514C, A513C or A1338C SNP occurred only in resistant strains and was not reported to occur in any strains susceptible to AMK, KAN and/or CAP, whereas the A1401G mutation alone was found to occur in up to 7% of CAP^S^ strains (Table 3). This suggests that multiple *rrs* mutations might be a highly specific predictor of injectable drug resistance when detected (i.e., never observed in susceptible strains).

**Table 7:** Cumulative Frequencies of Multiple Mutations within the *rrs* Gene among *Mycobacterium tuberculosis* Isolates Resistant or Susceptible to Amikacin (AMK), Kanamycin (KAN) and/or Capreomycin (CAP).

Mutation 1	Mutation 2	Drug Tested	#R Examined	#S Examined	#R with Mutation	#S with Mutation	Frequency of Mutation among R	Frequency of Mutation among S
A1401G	A514C	AMK	200	263	10	0	0.05	0.00
		KAN	269	236	10	0	0.04	0.00
		CAP	195	273	10	0	0.05	0.00
A1401G	A1338C	AMK	293	473	5	0	0.02	0.00
		KAN	404	405	5	0	0.01	0.00
		CAP	288	482	5	0	0.02	0.00
A1401G	A513C	AMK	196	260	1	0	0.01	0.00
		KAN	259	239	8	0	0.03	0.00
		CAP	172	259	1	0	0.01	0.00
G1484T	C1402A	AMK	443	624	0	1	0.00	0.00
		KAN	525	543	3	0	0.01	0.00
		CAP	434	637	1	0	0.00	0.00

R = Resistant isolates.

S = Susceptible isolates.

## Discussion

It is clear from the literature reviewed that *Mtb* mutations associated with injectable drug resistance are understudied compared to the mutations associated with resistance to first-line drugs [42]. Only the 1401 and 1484 positions of the *rrs* gene have been evaluated to any extent in injectable drug resistant isolates (n>600), while the remainder of the *rrs* and other resistance-associated genes (*tlyA* and the *eis* promoter) have each been evaluated in less than 400 drug resistant *Mtb* isolates. Only 20 injectable drug resistant isolates have been examined for *gidB* mutations. This review uncovered evidence to suggest that certain mutations outside the commonly-observed A1401G SNP, alone or in conjunction with *rrs* mutations, could help to detect some of the 25%–40% of AMK, KAN, and CAP resistant *Mtb* strains that do not appear to have an *rrs* mutation.

Future studies of *Mtb* resistant to AMK, KAN and CAP should always include a comprehensive sequence analysis of at least the entire four genes examined here, if not the entire *Mtb* genome. Additionally, a suitable number of injectable drug susceptible strains should be included in each study to build data on the potential specificity of each mutation. As the sensitivity of any mutation-based molecular diagnostic will be determined by both the strength of the association between mutation and phenotypic resistance *and* the frequency of the specific resistance-related mutation in the population, it is also crucial to establish both regional and global frequencies of these mutations to predict where and how to develop the most effective molecular diagnostics.

### Mutations as Markers of Phenotypic Resistance to the Injectable Drugs

Certain *rrs*, *tlyA*, *eis* promoter and *gidB* gene mutations with the potential to be both sensitive and specific predictors of phenotypic drug resistance were found frequently in drug-resistant strains and infrequently, or never, in drug-susceptible strains. Compared to INH-resistant *Mtb* strains (∼85% of which have *katG* and/or *inhA* promoter mutations) [43] and RIF resistant strains (∼97% of which have *rpoB* mutations) [44], phenotypic resistance to AMK, KAN and/or CAP was associated with a larger diversity of genes, each with lower cumulative frequencies.

The *rrs* A1401G SNP was reported in about 75% of CAP^R^ and AMK^R^ strains, and in less than 60% of KAN^R^ strains, and it was only found in 7% of strains susceptible to CAP, suggesting this mutation would be a very sensitive and specific marker for phenotypic resistance to AMK, a very sensitive but moderately specific marker for phenotypic resistance to CAP, and a moderately sensitive but very specific marker for resistance to KAN.

It appears that a single mutation, or even a set of mutations in a single gene, does not adequately predict phenotypic resistance to AMK, KAN and CAP. It is likely that a combination of different gene mutations for each injectable drug will be needed to best predict phenotypic resistance, as has been reported for the molecular detection of INH resistance [45].

Based on the published mutation frequencies we report here, KAN^R^ strains might be predicted with highest sensitivity and specificity using a combination of *rrs* mutations (e.g. A1401G and C517T) and *eis* promoter mutations (e.g. G-10A and C-14). Since *eis* promoter mutations reportedly occur almost exclusively in KAN^R^ strains with no 1401 *rrs* mutation [37], [39] it is likely that this approach will be considerably more sensitive than using the rrs 1401 mutation alone. CAP^R^ strains, on the other hand, appear to be best predicted by mutations in *rrs* gene (i.e., A1401G, C1402T, G1158T) possibly together with mutations such as C-12T in the *eis* promoter, which was reported in 5% of CAP^R^ strains and only 1% of CAP^S^ strains. Another useful marker might be the insertion at position 755 of the *tlyA* gene; however, future studies will be needed to confirm the frequency of this, and other, mutations in CAP^R^ strains worldwide. For AMK^R^ strains, a combination of the A1401G *rrs* SNP together with the *eis* promoter mutations C-14T or C-12T (reported in 9% and 6% of AMK^R^ strains) could increase sensitivity of resistance detection without decreasing specificity. There was not sufficient data to evaluate the potential role of double *rrs* mutations, but given that they were never reported in injectable drug susceptible *Mtb* strains, these mutations could significantly increase the positive predictive value of rapid diagnostics when found.

It is important to note that resistance to streptomycin (SM), an aminoglycoside with a similar mechanism to AMK and KAN [46], was not evaluated in this review as it is not as well-studied as the XDR-TB defining drugs. It is possible this exclusion may have confounded some of our findings regarding mutations in *gidB* and *rrs*. As SM is often administered together with AMK, KAN or CAP, and SM resistance has been associated with mutations in *gidB* [47] and the 500 region of *rrs* [48], that are not thought to cross-react with AMK, KAN or CAP resistance; it is possible that the associations we observed in these gene regions are a reflection of underlying, but unevaluated SM resistance rather than true associations with AMK, KAN and CAP resistance. Further study of these mutations in the absence of SM resistance could shed important light on the value of these mutations.

### Improving the Detection of AMK, KAN and/or CAP Resistance with Existing Molecular Diagnostics

In considering the implications of our findings, we focus upon three commercially available molecular diagnostics employed to detect resistance-related mutations in *Mtb* clinical isolates [49]: hybridization-based tests, pyrosequencing, and molecular beacon testing. Hybridization tests, such as the MTBDR*plus* and MTBDR*sl* line probe assays (LPAs) (Hain Life Sciences, Tübingen, Germany), are based on the hybridization of specific mutations in clinical strains to a probe that is complimentary to the mutated DNA. The MTBDR*plus/sl* assays are the only commercially available rapid molecular diagnostics currently in broad use for the detection of both MDR-TB and XDR-TB. They have shown great promise in field studies for the detection of MDR-TB [8], [50]–[60], but variable sensitivity (40%–100% depending on the number of strains examined) for detection of injectable drug resistance [9], [10], [29], [36], [61]. As the MTBDR*sl* LPA detects only mutations in the 1401, 1402 and 1484 positions of the *rrs* gene, the varying sensitivity of the test is in agreement with the apparent cumulative frequency of these mutations in the published literature. It would also seem from the very low cumulative frequencies of the *rrs* 1402 and 1484 mutations reported, that their inclusion in the MTBDR*sl* LPA as the only mutations outside of *rrs* 1401, might be limiting the potential sensitivity of this test for the detecting AMK, KAN and CAP resistance. Adding or replacing probes could potentially increase MTBDR*sl* sensitivity by as much as 10% without significantly decreasing specificity. In a recent evaluation study of the MTBDR*sl* test, the inclusion of mutations from a second gene, the *eis* promoter, increased field sensitivity for KAN^R^ detection by about 30% [36].

Pyrosequencing is a rapid sequencing method used to detect genetic mutations based on “sequencing by synthesis”. It has been established as a valid rapid method for detecting resistance-associated SNPs in MDR-TB strains [62]–[66], but only one small study reported on its use for XDR-TB detection [59]. A large, multinational field study of suspected M/XDR-TB patients in India, Moldova and South Africa [67] is currently evaluating the effectiveness of pyrosequencing for detecting resistance to AMK, KAN and CAP based on detection of mutations in the *rrs* gene between base pair 1397 through 1406. Increasing the number of mutations and genes currently being detected by pyrosequencing in this study could significantly improve the next iteration of the assay.

Beacon-type sequencing, or SNP genotyping, has shown excellent utility in the field [68]–[71] and is the basis of the GeneXpert assay that was recently endorsed by WHO for detecting RIF resistance in *Mtb* [72]. Unfortunately, this technology is still being developed for XDR-TB detection, and there is a risk that detection of second-line drug resistance may fall behind unless the study of the appropriate mutations is accelerated. Beacon technology is based on detection of short stretches of genes that are different from the wild type genome, rather than detecting point mutations like an LPA. Nonetheless, it could similarly be developed to detect a variety of mutations in short regions of the *rrs*, *eis* promoter, *tlyA*, and *gidB* genes if the limitations in beacon development for these regions can be overcome.

### Limitations

The cumulative frequencies we calculated were based on several assumptions, but the two we describe here are the most important. First, we assumed that all the mutations reported were independent of each other. If some isolates were misclassified as independent when they were in fact not, this could have caused an overestimation in our cumulative frequencies of that specific mutation. Every effort was made to ensure that the isolates and the mutations presented in one study were not also reported in another study. All manuscripts were carefully examined to identify isolates that were used in more than one study (e.g., same isolate origin, same authors or organization, etc.) and to the best of our knowledge, all isolates reported here are unique. A second potential source of misclassification error was in our use of the DST results as reported. For example, if an isolate was misclassified as resistant based on faulty DST data, when it was in fact susceptible, and it did not have the expected mutation, then we would have underestimated the cumulative frequency of that mutation among resistant isolates. To minimize the chances of such misclassification, we excluded manuscripts without explicit descriptions of their DST methods and clear definitions of what constituted a resistant or susceptible isolate using accepted DST drug concentrations and methodologies.

### Conclusion

It is clear that the *rrs* 1401 mutation alone was not found with sufficient frequency to detect more than 70–80% of global *Mtb* strains resistant to AMK and CAP, and 60% of strains resistant to KAN. While this is likely sufficient for a rapid screening test, future molecular diagnostics will need to include more mutations in more genes in order to accurately and sensitively detect resistance and cross-resistance to AMK, KAN and CAP for clinical decision-making purposes.

Other SNPs in the *rrs*, *eis* promoter, *tlyA* and *gidB* appear to be very promising markers for improving both sensitivity and specificity of detection of AMK, KAN and CAP resistance and cross-resistance, but it is likely that each drug will need to be considered independently (with independent mutation profiles) to maximize the diagnostic and clinical utility of future molecular diagnostics for these drugs.

## Supporting Information

Figure S1 PRISMA flowchart for systematic review.
(DOC)

Table S1 PRISMA checklist for systematic review.
(DOC)

## References

[1] Organization WH (2011). Global Tuberculosis Control.

[2] Organization WH (2011).

[3] Raviglione MC, Smith IM (2007). XDR tuberculosis–implications for global public health.. N Engl J Med.

[4] Organization WH (2010). Global Tuberculosis Control.

[5] Calver AD, Falmer AA, Murray M, Strauss OJ, Streicher EM (2010). Emergence of increased resistance and extensively drug-resistant tuberculosis despite treatment adherence, South Africa.. Emerg Infect Dis.

[6] Pfyffer GE, Bonato DA, Ebrahimzadeh A, Gross W, Hotaling J (1999). Multicenter laboratory validation of susceptibility testing of Mycobacterium tuberculosis against classical second-line and newer antimicrobial drugs by using the radiometric BACTEC 460 technique and the proportion method with solid media.. J Clin Microbiol.

[7] Shah NS, Richardson J, Moodley P, Moodley S, Babaria P (2011). Increasing drug resistance in extensively drug-resistant tuberculosis, South Africa.. Emerg Infect Dis.

[8] Hillemann D, Weizenegger M, Kubica T, Richter E, Niemann S (2005). Use of the genotype MTBDR assay for rapid detection of rifampin and isoniazid resistance in Mycobacterium tuberculosis complex isolates.. J Clin Microbiol.

[9] Kiet VS, Lan NT, An DD, Dung NH, Hoa DV (2010). Evaluation of the MTBDRsl test for detection of second-line-drug resistance in Mycobacterium tuberculosis.. J Clin Microbiol.

[10] Brossier F, Veziris N, Aubry A, Jarlier V, Sougakoff W (2010). Detection by GenoType MTBDRsl test of complex mechanisms of resistance to second-line drugs and ethambutol in multidrug-resistant Mycobacterium tuberculosis complex isolates.. J Clin Microbiol.

[11] Boehme CC, Nabeta P, Hillemann D, Nicol MP, Shenai S (2010). Rapid molecular detection of tuberculosis and rifampin resistance.. N Engl J Med.

[12] Watterson SA, Wilson SM, Yates MD, Drobniewski FA (1998). Comparison of three molecular assays for rapid detection of rifampin resistance in Mycobacterium tuberculosis.. J Clin Microbiol.

[13] Drobniewski FA, Wilson SM (1998). The rapid diagnosis of isoniazid and rifampicin resistance in Mycobacterium tuberculosis–a molecular story.. J Med Microbiol.

[14] Alcaide F, Coll P (2011). Advances in rapid diagnosis of tuberculosis disease and anti-tuberculous drug resistance.. Enferm Infecc Microbiol Clin.

[15] Magnet S, Blanchard JS (2005). Molecular insights into aminoglycoside action and resistance.. Chem Rev.

[16] Trnka L, Smith DW (1970). Proteosynthetic activity of isolated ribosomes of Mycobacteria and its alteration by rifampicin and related tuberculostatic drugs.. Antibiot Chemother.

[17] Blaschitz M, Hasanacevic D, Hufnagl P, Hasenberger P, Pecavar V (2011). Real-time PCR for single-nucleotide polymorphism detection in the 16S rRNA gene as an indicator for extensive drug resistance in Mycobacterium tuberculosis.. J Antimicrob Chemother.

[18] Feuerriegel S, Cox HS, Zarkua N, Karimovich HA, Braker K (2009). Sequence analyses of just four genes to detect extensively drug-resistant Mycobacterium tuberculosis strains in multidrug-resistant tuberculosis patients undergoing treatment.. Antimicrob Agents Chemother.

[19] Honore N, Marchal G, Cole ST (1995). Novel mutation in 16S rRNA associated with streptomycin dependence in Mycobacterium tuberculosis.. Antimicrob Agents Chemother.

[20] Johansen SK, Maus CE, Plikaytis BB, Douthwaite S (2006). Capreomycin binds across the ribosomal subunit interface using tlyA-encoded 2′-O-methylations in 16S and 23S rRNAs.. Mol Cell.

[21] Suzuki M, Funatsu T, Tanaka H, Usuda S (1998). YM866, a novel modified tissue-type plasminogen activator, affects left ventricular function and myocardial infarct development in dogs with coronary artery thrombi.. Jpn J Pharmacol.

[22] Alangaden GJ, Kreiswirth BN, Aouad A, Khetarpal M, Igno FR (1998). Mechanism of resistance to amikacin and kanamycin in Mycobacterium tuberculosis.. Antimicrob Agents Chemother.

[23] Maus CE, Plikaytis BB, Shinnick TM (2005). Molecular analysis of cross-resistance to capreomycin, kanamycin, amikacin, and viomycin in Mycobacterium tuberculosis.. Antimicrob Agents Chemother.

[24] Maus CE, Plikaytis BB, Shinnick TM (2005). Mutation of tlyA confers capreomycin resistance in Mycobacterium tuberculosis.. Antimicrob Agents Chemother.

[25] Kruuner A, Jureen P, Levina K, Ghebremichael S, Hoffner S (2003). Discordant resistance to kanamycin and amikacin in drug-resistant Mycobacterium tuberculosis.. Antimicrob Agents Chemother.

[26] Sugawara I, Zhang J, Li C (2009). Cross-resistance of Mycobacterium tuberculosis isolates among streptomycin, kanamycin and amikacin.. Indian J Exp Biol.

[27] Ioerger TR, Koo S, No EG, Chen X, Larsen MH (2009). Genome analysis of multi- and extensively-drug-resistant tuberculosis from KwaZulu-Natal, South Africa.. PLoS One.

[28] Jugheli L, Bzekalava N, de Rijk P, Fissette K, Portaels F (2009). High level of cross-resistance between kanamycin, amikacin, and capreomycin among Mycobacterium tuberculosis isolates from Georgia and a close relation with mutations in the rrs gene.. Antimicrob Agents Chemother.

[29] Hillemann D, Rusch-Gerdes S, Richter E (2009). Feasibility of the GenoType MTBDRsl assay for fluoroquinolone, amikacin-capreomycin, and ethambutol resistance testing of Mycobacterium tuberculosis strains and clinical specimens.. J Clin Microbiol.

[30] Evans J, Segal H (2010). Novel multiplex allele-specific PCR assays for the detection of resistance to second-line drugs in Mycobacterium tuberculosis.. J Antimicrob Chemother.

[31] Perdigao J, Macedo R, Malaquias A, Ferreira A, Brum L (2010). Genetic analysis of extensively drug-resistant Mycobacterium tuberculosis strains in Lisbon, Portugal.. J Antimicrob Chemother.

[32] Via LE, Cho SN, Hwang S, Bang H, Park SK (2010). Polymorphisms associated with resistance and cross-resistance to aminoglycosides and capreomycin in Mycobacterium tuberculosis isolates from South Korean Patients with drug-resistant tuberculosis.. J Clin Microbiol.

[33] Leung KL, Yip CW, Yeung YL, Wong KL, Chan WY (2010). Usefulness of resistant gene markers for predicting treatment outcome on second-line anti-tuberculosis drugs.. J Appl Microbiol.

[34] Khanna A, Raj VS, Tarai B, Sood R, Pareek PK (2010). Emergence and molecular characterization of extensively drug-resistant Mycobacterium tuberculosis clinical isolates from the Delhi Region in India.. Antimicrob Agents Chemother.

[35] Campbell PJ, Morlock GP, Sikes RD, Dalton TL, Metchock B (2011). Molecular detection of mutations associated with first- and second-line drug resistance compared with conventional drug susceptibility testing of Mycobacterium tuberculosis.. Antimicrob Agents Chemother.

[36] Huang WL, Chi TL, Wu MH, Jou R (2011). Performance assessment of the GenoType MTBDRsl test and DNA sequencing for detection of second-line and ethambutol drug resistance among patients infected with multidrug-resistant Mycobacterium tuberculosis.. J Clin Microbiol.

[37] Engstrom A, Perskvist N, Werngren J, Hoffner SE, Jureen P (2011). Comparison of clinical isolates and in vitro selected mutants reveals that tlyA is not a sensitive genetic marker for capreomycin resistance in Mycobacterium tuberculosis.. J Antimicrob Chemother.

[38] Sirgel FA, Tait M, Warren RM, Streicher EM, Bottger EC (2011). Mutations in the rrs A1401G Gene and Phenotypic Resistance to Amikacin and Capreomycin in Mycobacterium tuberculosis.. Microb Drug Resist.

[39] Zaunbrecher MA, Sikes RD, Metchock B, Shinnick TM, Posey JE (2009). Overexpression of the chromosomally encoded aminoglycoside acetyltransferase eis confers kanamycin resistance in Mycobacterium tuberculosis.. Proc Natl Acad Sci U S A.

[40] Sun Z, Zhang J, Zhang X, Wang S, Zhang Y (2008). Comparison of gyrA gene mutations between laboratory-selected ofloxacin-resistant Mycobacterium tuberculosis strains and clinical isolates.. Int J Antimicrob Agents.

[41] Camus JC, Pryor MJ, Medigue C, Cole ST (2002). Re-annotation of the genome sequence of Mycobacterium tuberculosis H37Rv.. Microbiology.

[42] Johnson R, Streicher EM, Louw GE, Warren RM, van Helden PD (2006). Drug resistance in Mycobacterium tuberculosis.. Curr Issues Mol Biol.

[43] Valvatne H, Syre H, Kross M, Stavrum R, Ti T (2009). Isoniazid and rifampicin resistance-associated mutations in Mycobacterium tuberculosis isolates from Yangon, Myanmar: implications for rapid molecular testing.. J Antimicrob Chemother.

[44] Telenti A, Imboden P, Marchesi F, Lowrie D, Cole S (1993). Detection of rifampicin-resistance mutations in Mycobacterium tuberculosis.. Lancet.

[45] Ramaswamy SV, Reich R, Dou SJ, Jasperse L, Pan X (2003). Single nucleotide polymorphisms in genes associated with isoniazid resistance in Mycobacterium tuberculosis.. Antimicrob Agents Chemother.

[46] Sharma D, Cukras AR, Rogers EJ, Southworth DR, Green R (2007). Mutational analysis of S12 protein and implications for the accuracy of decoding by the ribosome.. J Mol Biol.

[47] Wong SY, Lee JS, Kwak HK, Via LE, Boshoff HIM (2011). Mutations in gidB Confer Low-Level Streptomycin Resistance in Mycobacterium tuberculosis.. Antimicrobial Agents and Chemotherapy.

[48] Springer B, Kidan YG, Prammananan T, Ellrott K, Bottger EC (2001). Mechanisms of streptomycin resistance: selection of mutations in the 16S rRNA gene conferring resistance.. Antimicrob Agents Chemother.

[49] O'Grady J, Maeurer M, Mwaba P, Kapata N, Bates M (2011). New and improved diagnostics for detection of drug-resistant pulmonary tuberculosis.. Curr Opin Pulm Med.

[50] Miotto P, Piana F, Penati V, Canducci F, Migliori GB (2006). Use of genotype MTBDR assay for molecular detection of rifampin and isoniazid resistance in Mycobacterium tuberculosis clinical strains isolated in Italy.. J Clin Microbiol.

[51] Cavusoglu C, Turhan A, Akinci P, Soyler I (2006). Evaluation of the Genotype MTBDR assay for rapid detection of rifampin and isoniazid resistance in Mycobacterium tuberculosis isolates.. J Clin Microbiol.

[52] Somoskovi A, Dormandy J, Mitsani D, Rivenburg J, Salfinger M (2006). Use of smear-positive samples to assess the PCR-based genotype MTBDR assay for rapid, direct detection of the Mycobacterium tuberculosis complex as well as its resistance to isoniazid and rifampin.. J Clin Microbiol.

[53] Hillemann D, Rusch-Gerdes S, Richter E (2007). Evaluation of the GenoType MTBDRplus assay for rifampin and isoniazid susceptibility testing of Mycobacterium tuberculosis strains and clinical specimens.. J Clin Microbiol.

[54] Ling DI, Zwerling AA, Pai M (2008). GenoType MTBDR assays for the diagnosis of multidrug-resistant tuberculosis: a meta-analysis.. Eur Respir J.

[55] Lacoma A, Garcia-Sierra N, Prat C, Ruiz-Manzano J, Haba L (2008). GenoType MTBDRplus assay for molecular detection of rifampin and isoniazid resistance in Mycobacterium tuberculosis strains and clinical samples.. J Clin Microbiol.

[56] Evans J, Stead MC, Nicol MP, Segal H (2009). Rapid genotypic assays to identify drug-resistant Mycobacterium tuberculosis in South Africa.. J Antimicrob Chemother.

[57] Nikolayevskyy V, Balabanova Y, Simak T, Malomanova N, Fedorin I (2009). Performance of the Genotype MTBDRPlus assay in the diagnosis of tuberculosis and drug resistance in Samara, Russian Federation.. BMC Clin Pathol.

[58] Miotto P, Piana F, Cirillo DM, Migliori GB (2008). Genotype MTBDRplus: a further step toward rapid identification of drug-resistant Mycobacterium tuberculosis.. J Clin Microbiol.

[59] Kontsevaya I, Mironova S, Nikolayevskyy V, Balabanova Y, Mitchell S (2011). Evaluation of two molecular assays for rapid detection of mycobacterium tuberculosis resistance to fluoroquinolones in high-tuberculosis and -multidrug-resistance Settings.. J Clin Microbiol.

[60] Scott LE, McCarthy K, Gous N, Nduna M, Van Rie A (2011). Comparison of Xpert MTB/RIF with other nucleic acid technologies for diagnosing pulmonary tuberculosis in a high HIV prevalence setting: a prospective study.. PLoS Med.

[61] van Ingen J, Simons S, de Zwaan R, van der Laan T, Kamst-van Agterveld M (2010). Comparative study on genotypic and phenotypic second-line drug resistance testing of Mycobacterium tuberculosis complex isolates.. J Clin Microbiol.

[62] Zhao MC, Bao L, Wu YH, Long Y, Zeng XW (2004). [Study on the gene mutation of quinolone-resistant Mycobacterium tuberculosis isolated from Sichuan Province].. Sichuan Da Xue Xue Bao Yi Xue Ban.

[63] Djelouadji Z, Henry M, Bachtarzi A, Foselle N, Raoult D (2009). Pyrosequencing identification of Mycobacterium tuberculosis W-Beijing.. BMC Res Notes.

[64] Bravo LT, Tuohy MJ, Ang C, Destura RV, Mendoza M (2009). Pyrosequencing for rapid detection of Mycobacterium tuberculosis resistance to rifampin, isoniazid, and fluoroquinolones.. J Clin Microbiol.

[65] Isola D, Pardini M, Varaine F, Niemann S, Rusch-Gerdes S (2005). A Pyrosequencing assay for rapid recognition of SNPs in Mycobacterium tuberculosis embB306 region.. J Microbiol Methods.

[66] Jureen P, Engstrand L, Eriksson S, Alderborn A, Krabbe M (2006). Rapid detection of rifampin resistance in Mycobacterium tuberculosis by Pyrosequencing technology.. J Clin Microbiol.

[67] Wakata A, Miyamae Y, Sato S, Suzuki T, Morita T (1998). Evaluation of the rat micronucleus test with bone marrow and peripheral blood: summary of the 9th collaborative study by CSGMT/JEMS. MMS. Collaborative Study Group for the Micronucleus Test. Environmental Mutagen Society of Japan. Mammalian Mutagenicity Study Group.. Environ Mol Mutagen.

[68] Varma-Basil M, El-Hajj H, Colangeli R, Hazbon MH, Kumar S (2004). Rapid detection of rifampin resistance in Mycobacterium tuberculosis isolates from India and Mexico by a molecular beacon assay.. J Clin Microbiol.

[69] Nebenzahl-Guimaraes H (2009). Recent patents on DNA sequences and diagnostic methods for the identification and strain differentiation of Mycobacterium tuberculosis.. Recent Pat DNA Gene Seq.

[70] Stavrum R, Myneedu VP, Arora VK, Ahmed N, Grewal HM (2009). In-depth molecular characterization of Mycobacterium tuberculosis from New Delhi–predominance of drug resistant isolates of the ‘modern’ (TbD1) type.. PLoS One.

[71] Banerjee R, Allen J, Lin SY, Westenhouse J, Desmond E (2010). Rapid drug susceptibility testing with a molecular beacon assay is associated with earlier diagnosis and treatment of multidrug-resistant tuberculosis in California.. J Clin Microbiol.

[72] Van Rie A, Page-Shipp L, Scott L, Sanne I, Stevens W (2010). Xpert((R)) MTB/RIF for point-of-care diagnosis of TB in high-HIV burden, resource-limited countries: hype or hope?. Expert Rev Mol Diagn.

[73] Organization WH (2008). Guidelines for the programmatic management of drug-resistant tuberculosis.

[74] Kleeberg HHKH, HP (1980). Susceptibility testing.. Laboratory manual of tuberculosis methods.

[75] Kent PTaK, GP (1985).

[76] Kam KM, Sloutsky A, Yip CW, Bulled N, Seung KJ (2010). Determination of critical concentrations of second-line anti-tuberculosis drugs with clinical and microbiological relevance.. Int J Tuberc Lung Dis.

[77] Jureen P, Angeby K, Sturegard E, Chryssanthou E, Giske CG (2010). Wild-type MIC distributions for aminoglycoside and cyclic polypeptide antibiotics used for treatment of Mycobacterium tuberculosis infections.. J Clin Microbiol.

[78] Kekkaku (1997). Drug susceptibility testing of Mycobacterium tuberculosis: a proportion method using Ogawa egg medium.. Committee for Laboratory Services of the Japanese Society for Tuberculosis.

