# Supplementary figures and images for: Evaluation of Genetic Mutations Associated with Mycobacterium tuberculosis Resistance to Amikacin, Kanamycin and Capreomycin: A Systematic Review
Source: PLoS One. 2012 Mar 29;7(3):e33275. doi: 10.1371/journal.pone.0033275 (PMC3315572; doi:10.1371/journal.pone.0033275)

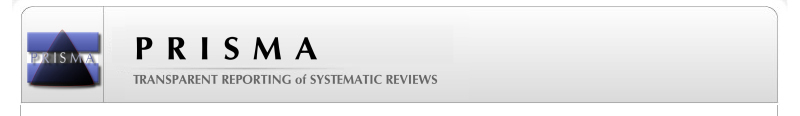
**PRISMA 2009 Flow Diagram**


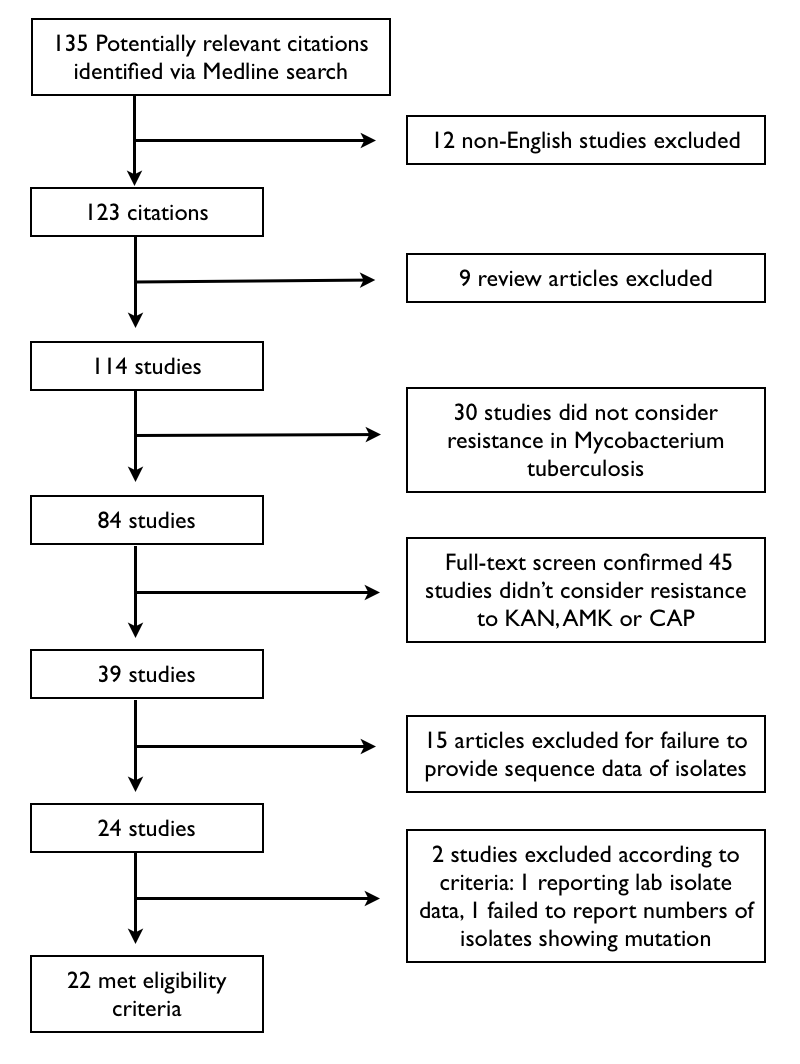


**Screening**

**Included**

**Eligibility**

**Identification**

Supplement: Figure S1 — PRISMA flowchart for systematic review. (DOC) [file pone.0033275.s001.doc]
